# Do single‐arm trials have a role in drug development plans incorporating randomised trials?

**DOI:** 10.1002/pst.1726

**Published:** 2015-11-26

**Authors:** Michael J. Grayling, Adrian P. Mander

**Affiliations:** ^1^ MRC Biostatistics Unit Hub for Trials Methodology ResearchCambridgeUK

**Keywords:** phase II clinical trial design, single‐arm, randomised two‐arm, optimal development plans

## Abstract

Often, single‐arm trials are used in phase II to gather the first evidence of an oncological drug's efficacy, with drug activity determined through tumour response using the RECIST criterion. Provided the null hypothesis of ‘insufficient drug activity’ is rejected, the next step could be a randomised two‐arm trial. However, single‐arm trials may provide a biased treatment effect because of patient selection, and thus, this development plan may not be an efficient use of resources. Therefore, we compare the performance of development plans consisting of single‐arm trials followed by randomised two‐arm trials with stand‐alone single‐stage or group sequential randomised two‐arm trials. Through this, we are able to investigate the utility of single‐arm trials and determine the most efficient drug development plans, setting our work in the context of a published single‐arm non‐small‐cell lung cancer trial. Reference priors, reflecting the opinions of ‘sceptical’ and ‘enthusiastic’ investigators, are used to quantify and guide the suitability of single‐arm trials in this setting. We observe that the explored development plans incorporating single‐arm trials are often non‐optimal. Moreover, even the most pessimistic reference priors have a considerable probability in favour of alternative plans. Analysis suggests expected sample size savings of up to 25% could have been made, and the issues associated with single‐arm trials avoided, for the non‐small‐cell lung cancer treatment through direct progression to a group sequential randomised two‐arm trial. Careful consideration should thus be given to the use of single‐arm trials in oncological drug development when a randomised trial will follow. Copyright © 2015 The Authors. *Pharmaceutical Statistics* published by JohnWiley & Sons Ltd.

## 1 Introduction

The development of a drug carries significant cost in terms of time 1 and money 2. Consequently, research into efficient designs for clinical trials is extremely active. Here, we focus on improving efficiency in phase II oncology trials. Because these early phase trials do not aim to provide definitive evidence of drug efficacy, much greater flexibility occurs in their design 3, and a healthy debate exists in terms of which is the best 4, 5, 6, 7, 8, 9, 10, 11, 12, 13.

Ultimately, these trials aim to make a go/no‐go decision and have classically taken the form of single‐arm trials 11, 14, with the most commonly used design being Simon's two‐stage 15. Indeed, a recent review indicated that over 20% of all phase II trials were Simon's designs 16. Single‐arm trials have been favoured because of their low expected sample size and ability to stop trials quickly when a drug has low activity. However, they do not have a causal interpretation because of a lack of randomisation. This means, trialists can introduce a selection bias leading to a biased treatment effect and a lack of confidence in a positive result. Subsequently, significant attention has been given lately to approaches for randomised phase II development plans 13.

However, randomised trials frequently require large sample sizes, and so the debate over their usage in phase II has continued. One way to potentially handle the issue of larger sample sizes though is to use a group sequential trial 17. These designs introduce a fixed number of interim analyses into a randomised trial, allowing early stopping for go or no‐go decisions, lowering the risk of exposure to inferior treatments.

With the debate over the optimal phase II development plan on‐going, a number of recent studies have compared the performance of single‐arm and randomised two‐arm trials under the influence of several factors 18, 19, 20, 21. It has been suggested that when possible randomised two‐arm trials should be used, even at the cost of higher sample sizes 19. Moreover, work has been conducted to explore which may be the best design for predicting phase III success 22, 23, also concluding it may often be preferable to use randomised two‐arm trials in phase II.

With the cost of late phase trials ever increasing, it could be argued that single‐arm trials alone should not be used as evidence for progression to phase III. Therefore, if a randomised trial is to be employed before a large‐scale confirmatory trial, we can question the role single‐arm trials have to play in the future. It may be more efficient to proceed directly to a randomised trial at phase II. Thus, in this work, we assess the benefits in terms of efficiency of a single‐arm followed by two‐arm trial development plan, in comparison to a sole two‐arm trial development plan. Reference priors 24 reflecting the opinions of ‘sceptical’ and ‘enthusiastic’ clinicians are used to quantify the probability that one of the development plans incorporating a single‐arm trial should be utilised. We set our analysis in the context of a completed trial on bavituximab plus paclitaxel and carboplatin for the treatment of advanced non‐small‐cell lung cancer 25.

## 2 Materials and Methods

### 2.1 Drug development scenario

We assume at the initiation of phase II that the maximum tolerated dose for an experimental treatment, or treatment combination, has been determined, and we are interested in initiating trials seeking to detect an efficacy signal. Specifically, we wish to compare this experimental intervention with the current standard (control) treatment, and determine if there is substantial activity against tumours to warrant further exploration. Additionally, we assume that the number of potential patients available for inclusion in the trial will not limit our design choices. Our ultimate goal is then to specify either a go decision of recommending further exploration, or a no‐go decision of recommending no further exploration of the experimental treatment. To this end, we consider six possible development plans, consisting of randomised two‐arm trials, with or without a preceding single‐arm stage. We assume a binary endpoint, because in this setting patient response, evaluated using the RECIST criterion 26, is usually the primary endpoint of interest. The work presented here could be extended to consider alternative endpoints, such as progression free survival, or to consider alternative development plans, but is not the focus of this paper. Moreover, we do not deal here with the case of rare diseases, for which there are few available participants, or the case when there is no presently available treatment. In these instances, alternate methods would be appropriate.

The hypotheses tested for the true response rate in the experimental treatment arm, *p*_*E*_, by the single‐arm trials are:

$$\begin{array}{cc} H_{0}^{s}:p_{E} & \leq p_{0},\\ H_{1}^{s}:p_{E} & \geq p_{1}>p_{0}. \end{array}$$

Here, *p*_0_ represents the fixed null response probability at which the drug will be considered to have insubstantial activity to be of further interest and can be chosen based on the historical response rate of the control treatment. Additionally, *p*_1_ represents the minimum desired response for the experimental treatment to constitute a clinically relevant benefit, worthy of further exploration. The type‐I error is controlled at *p*_*E*_=*p*_0_ to a level *α*_*s*_, and type‐II error at *p*_*E*_=*p*_1_ to *β*_*s*_.

The randomised two‐arm trials test the following hypotheses for the difference in the true response rate of the experimental and control treatment arms, *p*_*D*_=*p*_*E*_−*p*_*C*_:

$$\begin{array}{cc} H_{0}^{R}:p_{D} & \leq 0,\\ H_{1}^{R}:p_{D} & \geq p_{1}-p_{0}. \end{array}$$

Here, type‐I error is controlled at *p*_*D*_=0 to a level *α*_*R*_ , and type‐II error at *p*_*D*_=*p*_1_−*p*_0_ to a level *β*_*R*_.

In this work, the following six development plans are compared:

- DP1: A Simon's two‐stage $H_{0}^{S}$‐optimal single‐arm trial 15, 27, followed by a randomised two‐arm trial if the null hypothesis of the single‐arm stage, $H_{0}^{S}$ is rejected.
- DP2: A Simon's two‐stage $H_{0}^{S}$‐optimal single‐arm trial with early stopping for efficacy 27, followed by a randomised two‐arm trial if the null hypothesis of the single‐arm stage, $H_{0}^{S}$, is rejected.
- DP3: A single‐stage randomised two‐arm trial.
- DP4: A group sequential two‐arm trial, with 3 stages, and early go/no‐go stopping according to error spending methods 28 with the ‘rho‐family’ spending function 17.
- DP5: A group sequential two‐arm trial with interim analyses for no‐go decisions timed according to the sample sizes of each stage of the identified Simon's two‐stage design in DP1, followed by an additional final analysis for go/no‐go decisions timed to provide the correct power. Stopping boundaries and sample sizes are identified through error spending methods 28 with the ‘rho‐family’ spending function 17.
- DP6: A group sequential two‐arm trial with an interim analysis for a no‐go decision timed according to the total sample size of the identified Simon's two‐stage design in DP1, followed by two additional equally spaced analyses for go/no‐go decisions timed to provide the correct power. Stopping boundaries and sample sizes are identified through error spending methods 28 with the ‘rho‐family’ spending function 17.

These six development plans thus allow us to consider the most likely single‐arm, followed by randomised two‐arm designs, along with standard single‐stage and group sequential two‐arm designs, and finally, two group sequential designs that are comparable with the single‐arm incorporating plans in terms of their timed interim analyses. Therefore, they provide coverage of a wide array of possible designs for investigators.

For DP1–DP2, we declare a final go decision for the experimental treatment if both $H_{0}^{S}$ and $H_{0}^{R}$ are rejected, whereas for DP3–DP6, a go decision occurs when only $H_{0}^{R}$ is rejected (because $H_{0}^{S}$ is not tested for DP3–DP6). All development plans are designed to have equal development plan wide type‐I and type‐II error rates, *α* and *β*, under the ‘global null hypothesis’ (*H*_0_)*p*_*C*_=*p*_*E*_=*p*_0_ and ‘global alternative hypothesis’ (*H*_1_)*p*_*C*_=*p*_0_, *p*_*E*_=*p*_1_, respectively. This allows their efficiencies to be compared whilst controlling the operating characteristics to be equal. Specifically, to ensure the type‐I and type‐II error rates of DP1–DP2 are *α* and *β* across their possible two trials, we set the single‐arm and two‐arm stages to each have type‐I error rate $\alpha_{S}=\alpha_{R}=\sqrt{\alpha }$ and type‐II error rate $\beta_{S}\;=\;\beta_{R}\;=\;1\;-\;\sqrt{1\;-\;\beta }$. A pictorial description of the plans can be found in Supplementary Figure 1. Note that we do not consider optimal spending of the type‐I and type‐II errors between the two studies in DP1–DP2. Furthermore, this formulation ensures the type‐I error rate of DP3–DP6 is always less than or equal to *α* (strong control) in the region $p_{E}\leq p_{C}$. However, there will be instances when $p_{E}\leq p_{C}$ and DP1–DP2 have a type‐I error rate greater than *α*. If strong control is desired for these development plans *α*_*R*_ could be set to *α*. Finally, we will refer to the case *p*_*C*_=0.15, *p*_*E*_=0.41 as the ‘observed response rates’ (*O*)(see below).

**Figure 1:**
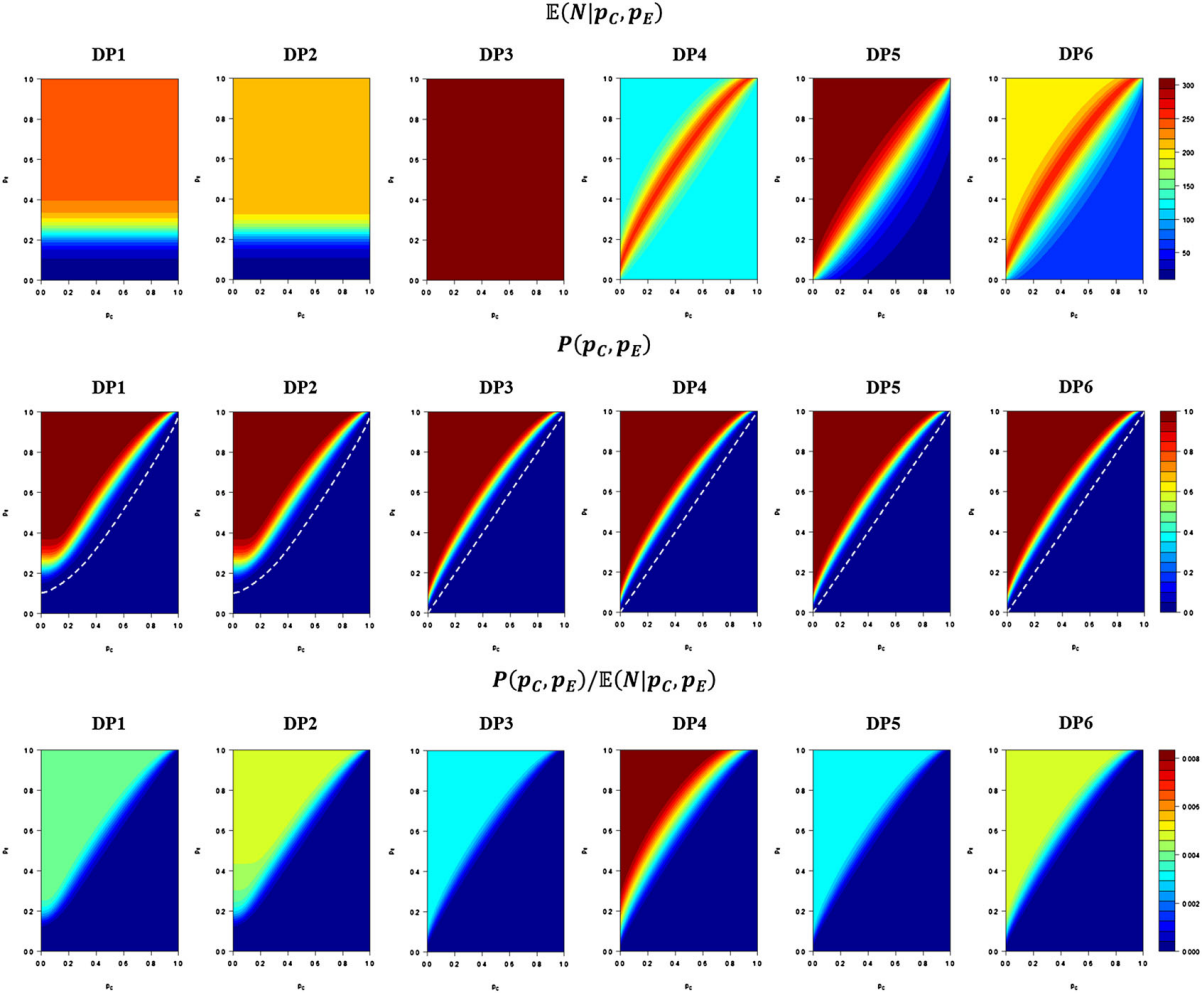
Development plan performances for *p*_0_=0.15, *p*_1_=0.3, *α* = 0.0025, *β* = 0.36. The expected sample size; $E(N|p_{C},p_{E})$, powerl; *P*(*p*_*C*_,*p*_*E*_), and ‘power per patient’; $P(p_{C},p_{E})/E(N|p_{C},p_{E})$, of the six development plans, across the complete possible range of true response rates in the control (*p*_*C*_) and treatment (*p*_*E*_) arms is depicted. The dotted white line in the *P*(*p*_*C*_,*p*_*E*_) plots indicates the contour *P*(*p*_*C*_,*p*_*E*_) = *α*.

### 2.2 Design performance

With the aforementioned specification, to determine designs for each of the development plans, we need only to specify *p*_0_, *p*_1_, *α* and *β*. Given values for these, the $H_{0}^{S}$‐optimal Simon's two‐stage designs were found using an exhaustive search over rejection/acceptance boundaries and sample sizes. For the single‐stage randomised two‐arm design, the minimum sample size required was calculated using the normal approximation to the difference of two binomial proportions 29. Finally, as noted in the preceding sections, the group sequential designs were identified using the error spending method of Lan and DeMets 28, using the ‘rho‐family’ spending function 17 with spending parameter 1. Further information on how these designs were determined can be found in the Supplementary Materials.

Following the development plan specifications, for each pair of possible true response rates in the control and experimental treatment arms, (*p*_*C*_,*p*_*E*_)ε[0,1] × [0,1], the probability that a go decision would be declared for each development plan (the power), $P(p_{C},p_{E})=P(\text{Reject}\;H_{0}|p_{C},p_{E})$, and the associated expected sample size, $E(N|p_{C},p_{E})$, was then determined. Full descriptions of the formulae used to compute *P*(*p*_*C*_,*p*_*E*_) and $E(N|p_{C},p_{E})$ are also provided in the Supplementary Materials. Note that we here simply compute the probability of rejecting *H*_0_, we do not make a distinction as to whether this was the correct decision.

### 2.3 Software

All analyses were performed using the software environment R 30. All code is available upon request from the authors.

### 2.4 Optimality criteria

In order to determine the optimal development plan, of the six considered, for each pair of true response rates, (*p*_*C*_,*p*_*E*_), several criteria were used. Classically, trial designs have been considered optimal subject to having the smallest expected sample at some minimum level of power. More recently however, the concept of ‘power per patient’ has been proposed as a possible new optimality criteria for clinical trial designs 31; allowing the two important factors of power and expected sample size to be combined into a single metric and designs with similar expected sample size but differing power to be compared more easily. Thus, the following four criteria were used here to determine the optimal design for each pair of values (*p*_*C*_,*p*_*E*_):

- OC1: $\min\{E(N|p_{C},p_{E})\}$,
- OC2: $I_{\left\{P(p_{C},p_{E})>1-\beta \right\}}\min\{E(N|p_{C},p_{E})\}$,
- OC3: $\max\{P(p_{C},p_{E})/E(N|p_{C},p_{E})\}$,
- OC4: $I_{\left\{P(p_{C},p_{E})>1-\beta \right\}}\max\{P(p_{C},p_{E})/E(N|p_{C},p_{E})\}$.

where *I* is the indicator function, which is 1 when there is at least 100(1 − *β*)*%* power and 0 otherwise. OC1 and OC3 allowed optimal development plans to be determined in zones of the parameter space where no plan met the minimal power criteria. Thus, OC1 simply declares the trial with the smallest expected sample size as optimal, whilst OC2 seeks the trial with the minimal expected sample size subject to having power of at least 100(1 − *β*)*%*. Further, OC3 searches for the maximal power per patient, with OC4 desiring maximal power per patient subject to having power of at least 100(1 − *β*)*%*.

### 2.5 Reference priors

Now, in order to determine the appropriate usage of single‐arm studies to a researcher, we compute probabilities that each of the six development plans is optimal after placing priors on the value of *p*_*E*_ with *p*_*C*_=*p*_0_. Explicitly, for each optimality criteria in turn, the prior probability of configurations (*p*_*E*_,*p*_*C*_=*p*_0_) for which each development plan is known to be optimal is taken to be the probability that this development plan should be employed. Specifically, we utilise sceptic and enthusiast beta distribution reference priors 24, to reflect believed distributions of likely values for *p*_*E*_. A sceptic was assumed to believe the distribution should be centred on *p*_0_, with only a 10% chance that *p*_*E*_>*p*_1_. Whilst an enthusiast would believe the distribution should be centred on *p*_1_, with only a 10% chance that *p*_*E*_<*p*_0_. Full details on how the beta distributions were determined are provided in the Supplementary Materials. Once the reference priors have been utilised to determine the probability each of the development plans should be used, the appropriate usage of the single‐arm incorporating development plans versus the randomised two‐arm only plans, according to each optimality criteria, can be quantified.

Note that the above‐mentioned does not involve any Bayesian analysis. Moreover, note that the distributions here are simply two chosen to represent possible (extreme) views of researchers. Much research exists on eliciting prior opinion, and a prior could be constructed to reflect the opinion of any researcher 32. Moreover, we have assumed here that historical data would allow the value of the control treatment to be known relatively accurately and have thus set *p*_*C*_=*p*_0_ when working with the reference priors. However, you need not make this assumption, if, for example, changes in supportive care indicate that the control treatment response rate may have changed.

### 2.6 Non‐small‐cell lung cancer trial analysis

A single‐arm study was recently completed to explore whether bavituximab in combination with paclitaxel and carboplatin could be a useful treatment regimen for advanced non‐small‐cell lung cancer 25. The trial had a type‐I error rate of 5%, and a type‐II error rate of 20%, for *p*_0_=0.15 and *p*_1_=0.3, employing a (non‐optimal) Simon's two‐stage design. Forty‐nine patients were recruited, and an objective response rate of 41% was observed, based on one complete response and 19 partial responses. Consequently, the null‐hypothesis of the trial was rejected, and a randomised trial of this regimen has now begun, comparing the performance of bavituximab with paclitaxel and carboplatin, to paclitaxel and carboplatin alone.

Taking *p*_0_=0.15, *p*_1_=0.3, with *α* = 0.0025 and *β* = 0.36 (such that *α*_*S*_=0.05 and *β*_*S*_=0.2 for DP1–DP2, as in the completed trial), we explore the efficiency of our six development plans in the context of determining the efficacy of bavituximab with paclitaxel and carboplatin. Moreover, a recently completed phase III trial estimated the response rate of paclitaxel and carboplatin alone for non‐small‐cell lung cancer to be 15% 33. Thus, we are able to confidently make the assumption that *p*_*C*_=*p*_0_ and quantify how likely as a sceptic or enthusiast you should utilise a single‐arm trial for this experimental treatment. Recommendations on how appropriate the investigators use of a single‐arm trial was can then be made.

## 3 Results

### 3.1 Development plan specifications

The design of each development plan was determined for *α* = 0.0025, *β* = 0.36, *p*_0_=0.15 and *p*_1_=0.3 and is presented in Table 1. The Simon's two‐stage designs, with and without early efficacy stopping, both required a maximum sample size of 55 patients and a first stage sample size of 19 patients. These designs would potentially, for DP1–DP2, be followed by a single‐stage randomised two‐arm trial of 186 patients. DP3, the sole single‐stage randomised two‐arm trial, required 302 patients. DP4 required 120 patients for each stage, whilst DP5 as stated earlier, times interim analyses for futility after 20 and 56 patients to correspond with the identified Simon's two‐stage design, with a final analysis after 310 patients. Finally, DP6 required slightly more patients, timing analyses after 56, 202 and 346 patients. All six development plans can be observed to have the desired operating characteristics under *H*_0_ and *H*_1_.

**Table 1:** Development plan specifications and operating characteristics.

		Randomised two‐arm sample Size									
	Simon's two‐ stage design	*n*_1_	*n*_2_	*n*_3_	max *N*	$E(N|H_{0})$	*P*(*H*_0_)	$E(N|H_{1})$	*P*(*H*_1_)	E(N|O)	*P*(*O*)
DP1	3/19 12/55	186	N/A	N/A	241	39.2	0.0024	199.1	0.6413	236.6	0.9735
DP2	(3 7)/19 12/55	186	N/A	N/A	241	39.3	0.0024	192.7	0.6419	216.9	0.9736
DP3	N/A	302	N/A	N/A	302	302.0	0.0025	302.0	0.6427	302.0	0.9929
DP4	N/A	120	240	360	360	146.7	0.0025	252.7	0.6479	176.7	0.9899
DP5	N/A	20	56	310	310	199.8	0.0025	292.6	0.6417	307.1	0.9844
DP6	N/A	56	202	346	346	146.6	0.0025	256.8	0.6424	213.2	0.9872

The identified designs for each of the four development plans are displayed. In addition, the probability of determining a go decision under *H*_0_; when *p*_*C*_=*p*_*E*_=*p*_0_, and under *H*_1_; *p*_*C*_=*p*_*E*_=*p*_1_ is displayed, along with the expected sample sizes in these scenarios and for the observed response rates in the conducted trial, *O*; *p*_*C*_=0.15, *p*_*E*_=0.41. Expected sample sizes are given to 1 decimal place, and powers to 4 decimal places.

### 3.2 Development plan performance

Figure 1 shows the expected sample size, power and power per patient of each of the development plans across all possible values of true response rates in the control and treatment arms. Small expected sample sizes for the development plans incorporating a single‐arm trial (DP1–DP2) exist for low levels of *p*_*E*_. This would be expected given the drug would be unlikely to reach the randomised stage. Additionally, a slightly lower expected sample size can be seen when early go stopping is incorporated into the Simon's two‐stage design (DP2), than over its exclusion (DP1), as would be anticipated. Expected sample sizes drop substantially for *p*_*E*_≫*p*_*C*_, or *p*_*E*_≪*p*_*C*_, in the group sequential design DP4, as the trial would likely stop early. Similarly, sample sizes drop for DP5–DP6 when *p*_*E*_≪*p*_*C*_ due to the presence of early no‐go stopping throughout, whilst they have larger expected samples when *p*_*E*_≫*p*_*C*_ owing to the fact that early go stopping is only present at latter interim analyses.

Power can be seen to be comparable across all development plans with the only noticeable differences around the region where $p_{C}\approx p_{E}$, and for small *p*_*C*_ and *p*_*E*_ in the single‐arm incorporating plans (DP1–DP2).

Finally, notable differences can be seen in the power per patient possessed by each development plan. Whilst all six development plans have low values when *p*_*E*_≪*p*_*C*_, as would be expected, DP4 has substantially higher values for *p*_*E*_≫*p*_*C*_.

### 3.3 Optimal development plans

**Figure 2:**
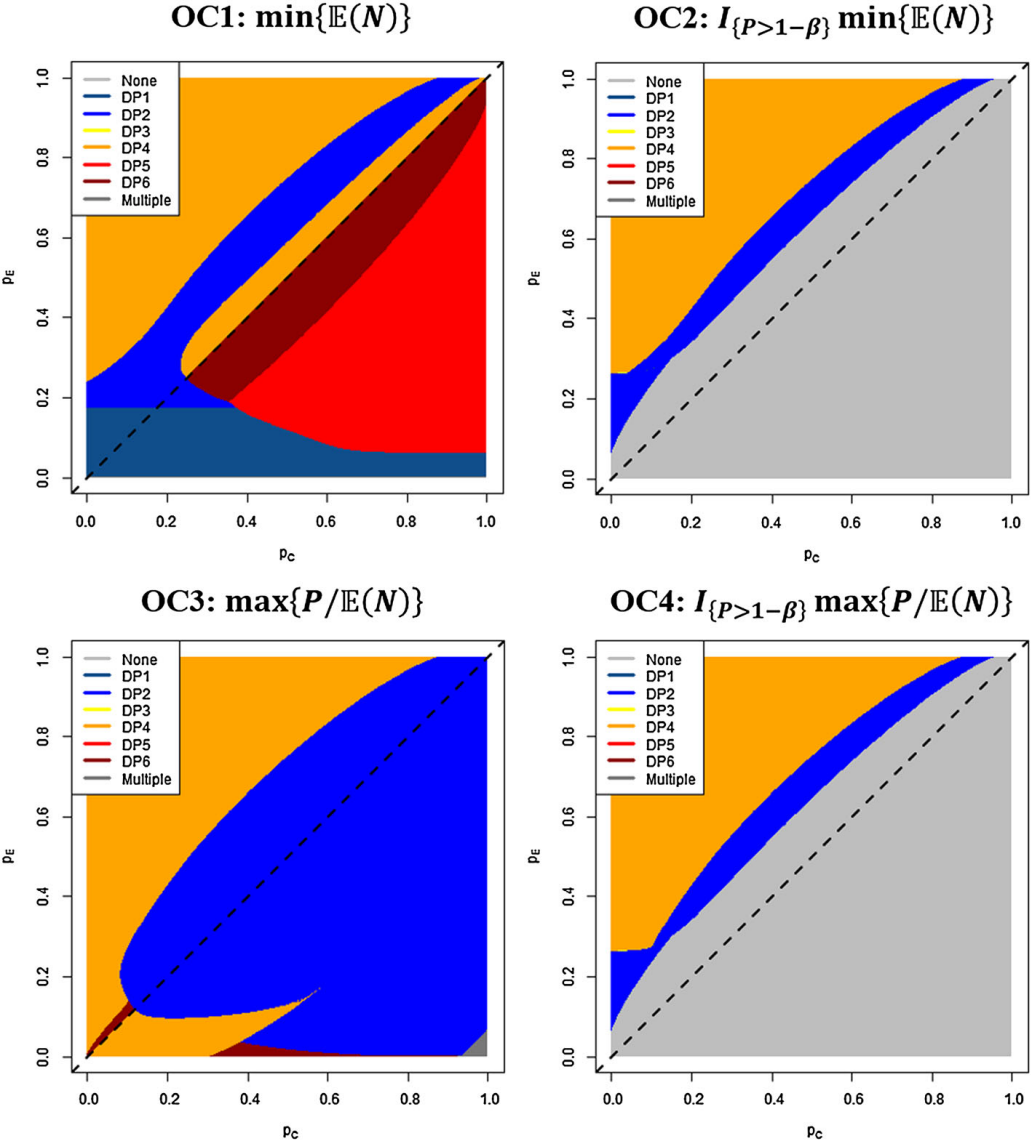
Optimal development plans. For the four optimality criteria (OC1–OC4), the optimal development plan is provided at each point in the parameter space [0,1] × [0,1].

Utilising the computed development plan performances, and our four optimality criteria, the optimal development plans for each pair of values (*p*_*C*_,*p*_*E*_)ε[0,1] × [0,1] were determined and are displayed in Figure 2. Summary information for *H*_0_, *H*_1_ and *O* is also provided in Table 1.

For the global null hypothesis *H*_0_, DP1 is found to be optimal under OC1 and DP2 optimal under OC3. In particular, the expected sample sizes of DP1–DP2 are substantially smaller here (39.2 and 39.3, respectively) in comparison to DP3–DP6 (302.0, 146.7, 199.8 and 146.6, respectively).

For the global alternative hypothesis *H*_1_, DP2 is optimal under all four optimality criteria. Here though, the sample sizes are more comparable (for example, 192.7 for DP2 in comparison with 252.7 for DP4). Moreover, for the observed response rates *O*, DP4 was optimal under all four optimality criteria, with an expected sample size of 176.7 in comparison with the next best performing DP6 with an expected sample size of 213.2.

Looking across the full parameter space [0,1] × [0,1], and the optimality criteria, we observe very few areas in which DP1 is optimal. Indeed, it only performs best for OC1 when *p*_*E*_ is small. Moreover, we observe no instance in which DP3 is optimal. For OC1, we observe large portions of the region $p_{E}\leq p_{C}$ in which DP5 and DP6 are optimal. For OC2 and OC4, where minimal power constraints are enforced, large regions where no development plan meets the requirements can be observed. In general, when an optimal development plan exists, it is either DP2 or DP4, and we observe many areas of the parameter space in which single‐arm incorporating plans are not optimal. Specifically, DP4 is optimal in the regions where *p*_*E*_≫*p*_*C*_, whilst DP2 performs better under OC3 for *p*_*E*_≪*p*_*C*_.

### 3.4 Sceptic and enthusiast development plan distributions

**Figure 3:**
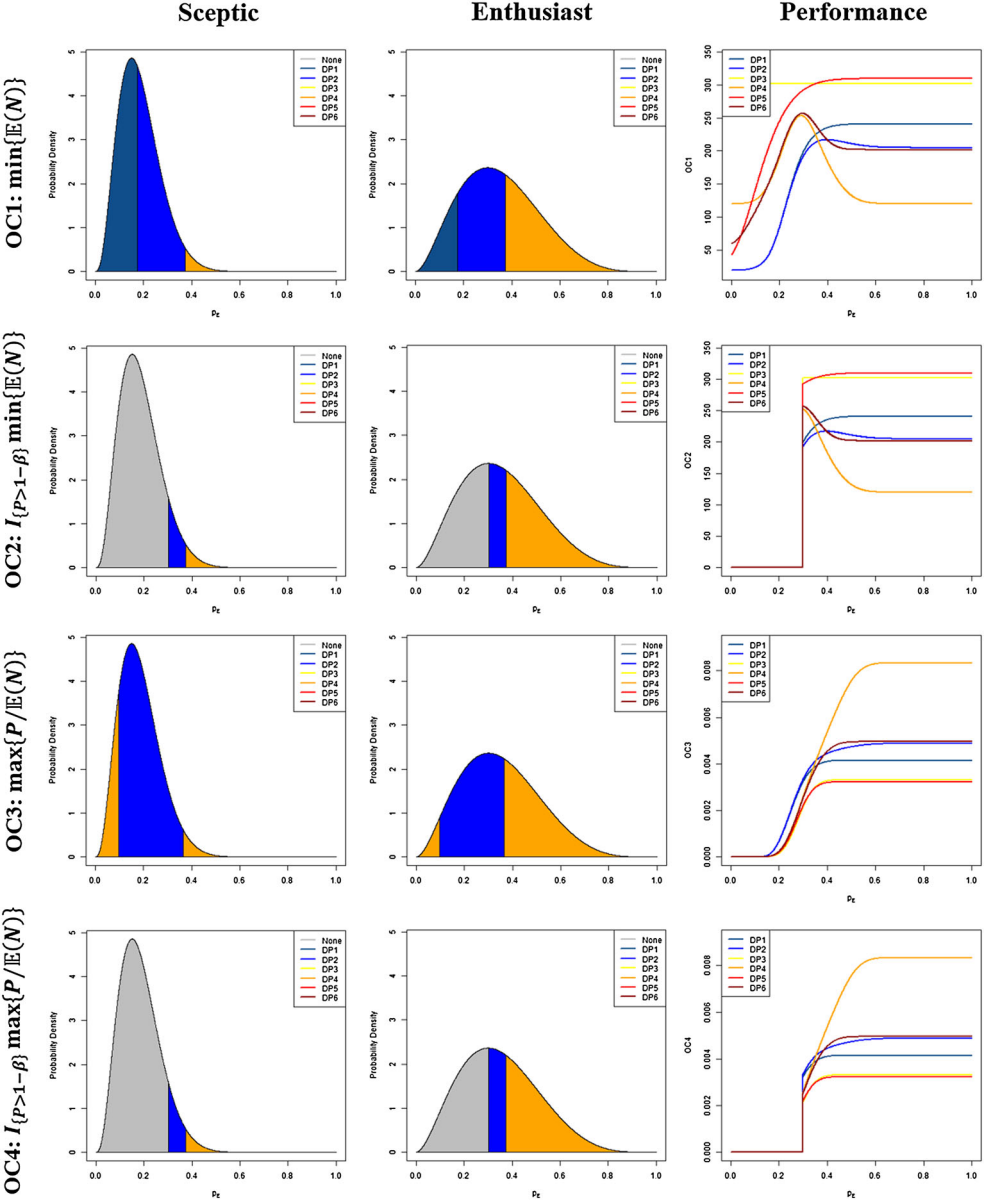
‘Sceptic’ and ‘Enthusiast’ development plan distributions. Beta distributions depict the optimal development plans according to the optimality criteria (OC1–OC4), and being either a ‘sceptic’ or an ‘enthusiast’. Additionally, the performance of each development plan for each optimality criteria, across values of *p*_*E*_, is displayed.

Figure 3 depicts the reference priors and the associated regions of values for *p*_*E*_ in which a sceptic or enthusiast should utilise each development plan, along with the performance of each development plan, for each optimality criteria. From these distributions, the probabilities of using a single‐arm trial (i.e. DP1–DP2), not using a single‐arm trial (i.e. DP3–DP6), and there being no optimal development plan, according to each optimality criteria, were computed and are shown in Table 2.

**Table 2:** ‘Sceptic’ and ‘enthusiast’ probabilities of utilising a single‐arm trial.

	Sceptic			Enthusiast		
	P(C)	P(S)	P(T)	P(C)	P(S)	P(T)
OC1: min{E(N)}	0.000	0.967	0.033	0.000	0.557	0.443
OC2: $I_{\left\{P>1-\beta \right\}}\min\{E(N)\}$	0.898	0.068	0.033	0.410	0.147	0.443
OC3: max{P/E(N)}	0.000	0.798	0.202	0.000	0.502	0.498
OC4: $I_{\left\{P>1-\beta \right\}}\max\{P/E(N)\}$	0.898	0.064	0.038	0.410	0.131	0.458

The probability of there being no optimal development plan under each optimality criteria (*C*), of a single‐arm trial utilising development plan being optimal (*S*), and of a non‐single‐arm trial utilising development plan being optimal (*T*) is shown for each optimality criteria, and according to being either a sceptic or enthusiast. All probabilities are given to 3 decimal places.

It is clear that as *p*_*E*_ increases, for all four optimality criteria, DP4 becomes the clear optimal design. However, for smaller values of *p*_*E*_, the performance between the development plans is more comparable.

We see that seeking to minimise the expected sample size (OC1), the usage of a single‐arm trial can be seen to outperform the two‐arm only development plans as a sceptic or an enthusiast (0.967 and 0.557 to 0.033 and 0.443, respectively), although the appropriate use probabilities are substantially closer as an enthusiast. In contrast, when power constraints are added to this criterion (OC2), the two‐arm only development plans are far more likely to be optimal as an enthusiast (0.443 to 0.147), but slightly less likely as a sceptic (0.033 to 0.068).

Similar patterns are observed for the criteria based on power per patient (OC3–OC4), where for OC3 the single‐arm incorporating plans are preferred as either a sceptic or an enthusiast, whilst for OC4, the two‐arm only plans are greatly favoured as an enthusiast but are less likely to be optimal as a sceptic.

## 4 Discussion

Future progress in cancer therapy can be accelerated by using better drug development plans in phase II. In this area a long‐standing debate exists over the use of single‐arm trials. Recent work has suggested that when the available patient population allows so, a randomised two‐arm trial may be preferable to a single‐arm trial 19. But, proponents of single‐arm designs may continue to favour their use; employing them prior to randomised trials, and citing their ability to identify poorly performing experimental treatments at low sample size. However, this may be less efficient than proceeding directly to a randomised two‐arm trial. We therefore considered whether a randomised two‐arm trial alone is preferable to initiating phase II with a single‐arm trial, before potentially proceeding to a randomised study. We utilised a published non‐small‐cell lung cancer trial to demonstrate the applicability of our analysis to the design of a real development plan. Through this, we were able to demonstrate that planning a complete development plan in advance allows the suitability of single‐arm trials in modern oncological drug development plans to be determined according to prior clinician opinion.

From our work, it is clear that for the majority of true response rates in the experimental and control arms, incorporating single‐arm trials is not optimal (Figure 2), additionally, it is clear that a single‐stage randomised two‐arm trial is never advisable. Often group sequential designs seem to be the most advisable approach. However, the regions in which the single‐arm incorporating development plans performed best frequently correspond to more probable regions of response rates; around the global null and alternative hypotheses, suggesting their use may be advisable.

Therefore, in order to more accurately determine the appropriate use of single‐arm trials within development plans, we placed reference priors on the possible response rate in the experiment arm to reflect possibly likely values according to clinician opinion. We found that the sceptic and enthusiast reference priors supported our observations above and below, indicating that in some cases the use of single‐arm trials remains the best course of action. However, it is interesting that for all optimality criteria, even as a sceptic who would be expected to favour single‐arm trials, there was always at least a 3% chance that development plans only consisting of randomised trials should be preferred.

It is a sad fact that the majority of treatments entering phase II are unlikely to be active. If a clinician believes this is to be the case then they will most probably wish to minimise the expected sample size. It is clear that for this optimality criteria (OC1) as a sceptic, or even as an enthusiast, the single‐arm incorporating plans should be utilised. Thus our analysis corroborates claims that there are situations in which single‐arm trials are applicable 7, 8, 9, 10, 34, 35. Even if the use of randomised trials at phase II continues to increase, single arm trials could have a role to play. However, the single‐arm and two‐arm stages of the development plan must be considered in advance, and even then simply utilising a randomised group sequential design may often be more efficient.

Moreover, one measure of optimality is unlikely to satisfy all scenarios and clinicians. Therefore, we compared results under three other criteria, which provided additional insight as to the appropriate use of single‐arm trials. Indeed, for OC2 where a minimal power constraint is enforced, we found that single‐arm trials would be preferred by a sceptic but not by an enthusiast. Incorporating this minimum power constraint reflects the instance in which the experimental treatment is efficacious, and it could easily be argued that a trial should only be conducted if it is believed that the treatment will show enough activity to warrant further exploration. Thus, the conclusion that it may often be useful to proceed directly to a group sequential trial could well be made.

Additionally, we explored the performance of the six development plans under the power per patient criterion, which considers the combination of power and expected sample size. Although it is a relatively new optimality criteria, its results are still of great interest. Analysis without a power constraint (OC3) revealed that both sceptics and enthusiasts should prefer the use of single‐arm trials. However, this criterion could potentially be questioned because many would desire minimal power per patient in the region $p_{E}\leq p_{C}$. This can be resolved through the addition of a minimal power requirement (OC4), in which case, similarly to OC2, it is clear that the use of single‐arm trials depends upon your standpoint as a sceptic or enthusiast. However, as an enthusiast, the randomised two‐arm only plans outperform the single‐arm incorporating ones by the largest margin of any optimality criteria. Thus it seems that if maximal value is desired in terms of the power per patient, investigators positive in the activity of their experimental treatment should certainly utilise a stand‐alone group sequential design at phase II.

Furthermore, for the non‐small‐cell lung cancer trial, the use of our analysis could have suggested a substantial probability in favour of not using a single‐arm trial in their development plan. Given their strong trial results, they may have avoided the use of what now appears to have been a less efficient development plan. Consequently, the speed of this drug's development could have been enhanced, and notable savings (up to 25% under the observed response rates) could have been made to the expected sample size.

As discussed earlier, several possible extensions to our work are evident, such as the exploration of alternative possible endpoints and development plans. Moreover, we have assumed that the control response rate would be known accurately. If this is not the case, investigators will need to alter the explored designs to account for possible heterogeneity in the control response rate. Alternatively, an additional prior of likely values could be placed on *p*_*C*_. In addition, we have assumed in DP1–DP2 that the information gained from the single‐arm trial will be disregarded in the design of the randomised two‐arm stage. This may well be the most likely practice as we are not aware of any published articles utilising historical data in such a manner in their primary analysis. However, such designs that take in to account the data from the single‐arm stage would be more efficient, and thus, exploration of such methods could prove interesting. For information on how to compute such designs, we refer the reader to 24, 36. Finally, although we have noted the problems associated with selection bias in single‐arm trials, we have not actually addressed this problem when assessing the efficiencies of the six development plans. Simulation studies could allow an investigator to analyse this accurately, although it seems likely that the performance of DP1–DP2 would worsen, and thus the use of single‐arm trials found to be even less favourable than they already appear to a clinician confident in the activity of their experiment treatment.

Now, it has already been noted that for the trial scenario explored here it may often be unwise to proceed to phase III on the evidence of single‐arm trials alone, owing to the issues of bias associated with them. Moreover, we have demonstrated that incorporating single‐arm trials in a preplanned manner before a randomised two‐arm trial will often be an inefficient use of patient resources if you believe a new treatment will be efficacious. In conclusion, whilst single‐arm trials certainly have a role to play still in phase II and although the design of any drug development plan should always be carefully selected and justified based on expert knowledge and the specific drug at hand, it seems reasonable that randomised only group sequential development plans in phase II should increasingly be a common practice. This could prove to be useful in improving the speed of drug development.

## Supporting information

Supporting info item

Supporting info item
