# Supplementary material for: Do single‐arm trials have a role in drug development plans incorporating randomised trials?
Source: Pharm Stat. 2015 Nov 26;15(2):143–51. doi: 10.1002/pst.1726 (PMC4855632; doi:10.1002/pst.1726)
Supplement: Supplementary file 1 — Supporting info item [file PST-15-143-s001.doc]

**Appendix**

**Development plan specification**

Recall that the single-arm trials in DP1-DP2 are designed to test:


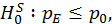


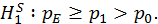


In what follows we assume that the designs desire to control the type-I error to some level
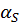
, at
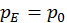
, and type-II error to some level
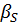
, at
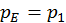
.

Moreover, the randomised two-arm trials in DP1-DP6 test the following hypotheses for the difference in the response rates of the experimental and control treatment arms,
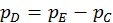
:


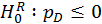


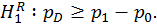


As above, in what follows we assume that the designs desire to control the type-I error to some level
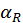
, at
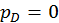
, and type-II error to some level
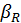
, at
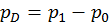
.

Now, for a Simon two-stage design without early stopping for a go decision, an index of “
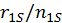

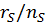
” is set, based upon an exhaustive search of all possible options up to some pre-set maximal value for
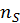
. The optimal index is chosen to be the one with the lowest expected sample size under the null hypothesis
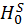
 that also meets the desired type-I and type-II error criteria,
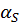
 and
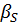
. Here
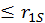
 responders out of the
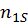
 patients recruited at the first stage results in early termination on no-go grounds. However,
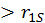
 responders results in a recruitment of
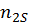
 further patients such that
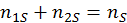
. Then,
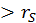
 responders out of the total
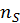
 patients results in rejection of
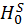
. Specifically, the following function is used to compute the probability that
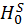
 is not rejected when
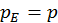
, for each possible “
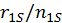

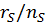
”:


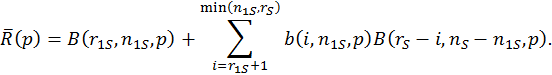


Here,
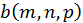
 and
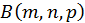
 are the density and cumulative density functions of a
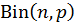
 variable. We then require
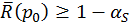
 and
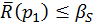
. Over the set of designs for which this is true, the optimal one is then that which minimises the expected sample size under
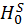
:


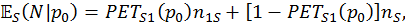


where
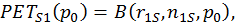
 is the probability of early termination.

With early stopping for a go decision added to the Simon two-stage design, an index of “
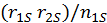

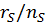
” is prescribed, after a search analogous to the one discussed above. Analysis proceeds as for the former case, except at the first stage if there are
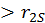
 responders in the first
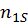
 patients, which would result in early termination to reject
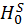
. Here, our function for determining designs conforming to type-I and type-II error criteria is:


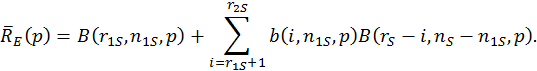


Here, we require
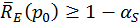
 and
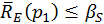
. Then, our optimality criteria here is:


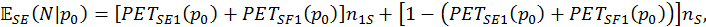


where
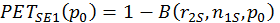
and
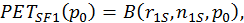
 are the probabilities of stopping for go and no-go decisions under
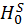
, respectively.

Now, the single-stage and group sequential randomised two-arm designs, are indexed by values for
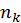
,
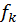
 and
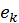
,
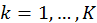
, where
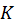
 is the maximal number of stages for the trial.
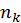
 denotes the number of patients who have been recruited at analysis
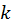
, the
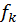
 correspond to the lower (futility) boundaries for no-go decisions and the
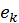
 correspond to the upper (efficacy) boundaries allowing stopping for a go decision. In all cases
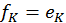
 in order to ensure the null hypothesis
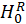
 is either accepted or rejected at the final analysis. As stated in the paper, the values for the
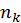
,
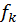
 and
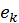
 of each trial are determined using the normal approximation to the binomial distribution. Our standardised test statistic at analysis
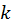
 is thus:


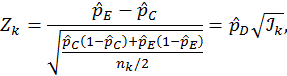


where we denote our information at stage
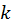
 by
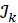
. Jennison and Turnbull (1999) state that the
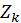
 follow what they call the canonical distribution:


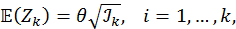


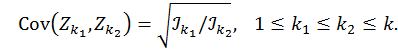


Here
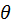
 is the true difference in treatment effects; and thus is taken as 0 for determining the type-I error rate, and
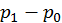
 for determining the type-II error rate.

To find the values for the
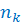
,
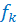
 and
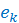
 of each randomised design (specified by values of
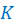
,
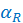
 and
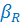
), we utilise the error spending approach of Lan and DeMets (1983) with the rho-family spending function (Jennison and Turnbull, 1999) for the type-I and type-II errors:


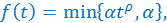


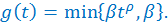


For all of the randomised two-arm designs we took
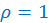
 as an example value, and in all cases final identified values for the
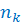
 are rounded up appropriately to the nearest even integers. For the single-stage randomised two-arm designs of DP1-DP3 we took
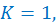
 whereas for DP4-6 we took
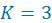
, with differences provided through the timing of interim analysis and allowed reasons for early stopping. Specifically, for DP4 we time the interim analysis to be equally spaced and allow early stopping for both go and no-go decisions, with no pre-set values for any of the
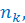

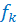
 or
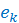
,
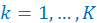
. For DP5 however we pre-set
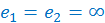
 and
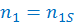
,
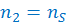
 to prevent early go stopping, and time the first two interim analysis according to the sample sizes required by the Simon two-stage design of DP1 (thereby creating a randomised two-arm only design as akin to DP1 as possible). Finally, for DP6 we pre-set
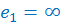
 and
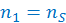
, equally spacing the timing of the remaining two interim analyses (thereby creating a randomised two-arm only design approximately half way between DP1 and a conventional group sequential design with 3 stages).


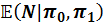
 **and**
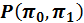
 **computation**

The following functions detail how expected sample size and power is computed for each development plan for a given pair of values
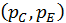
, of the true response rates on the control and experimental treatments. It is important to recall throughout that the values of
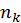
, and (as defined below) are development plan dependent, as well as the values of the indexes of the Simon two-stage designs.

For all of the randomised two-arm trial designs in DP1-DP6, given a pair of values , the probability of crossing an efficacy or futility bound at each analysis point can be determined by numerical integration as described in Chapter 19 of Jennison and Turnbull (1999). We denote below the probability of crossing an upper (efficacy) bound at stage by , and similarly the probability of crossing a lower (futility) bound at stage by .

Now, for DP1; the Simon two-stage design without early go stopping, followed by a randomised two-arm trial if the null hypothesised is rejected at the Simon step, we compute the required statistics using the following functions:

Here denotes the probability of termination at the first stage of the Simon step, and the probability of termination at the second stage of the Simon step having not terminated at the first step.

For DP2; the Simon two-stage design with early stopping for a go decision, followed by a randomised two-arm trial if the null hypothesised is rejected at the Simon step, we employ the following functions:

Here and denote the probability of termination at the first stage of the Simon step on go and no-go grounds respectively. and denote the probability of, and probability of not, terminating at the second stage of the Simon step for futility reasons having not terminated at the first stage.

For DP3-DP6, we make use of the following functions:

**Beta distribution computation**

To find the parameters (and for the ‘sceptic’ distribution, and and for the ‘enthusiast’ distribution) describing the shape of our beta distributions an optimisation step was used with the following criteria to meet:

Here the first conditions ensured the resulting Beta distributions were centred in the correct locations (i.e. took their maximal density there), whilst the latter conferred a % chance that the random variable reflecting the response rate in the treatment arm, , lied in the correct tail. The optimisation was performed by searching over possible values for and . The corresponding required value for and for the first condition to be satisfied was determined for each and . With these values set the two tail probabilities, and , could be determined and compared to . Finally, the parameters implying tail probabilities closest to were chosen as the optimal ones. In the paper, we take .

With the beta distributions set, the probability that a ‘sceptic’ or ‘enthusiast’ should employ each of the different development plans could be returned by integrating over the regions in which that development plan was optimal. For example, if DP4 was optimal in the region then the probability it should be employed by a ‘sceptic’ would be given by:

where is the probability density function of a distribution. Finally, the appropriate usage of single-arm incorporating designs for a ‘sceptic’ or ‘enthusiast’ was determined by summing the probabilities for DP1-DP2, and similarly for the non-single-arm incorporating development plans.
