# Supplementary figures and images for: Do single‐arm trials have a role in drug development plans incorporating randomised trials?
Source: Pharm Stat. 2015 Nov 26;15(2):143–51. doi: 10.1002/pst.1726 (PMC4855632; doi:10.1002/pst.1726)

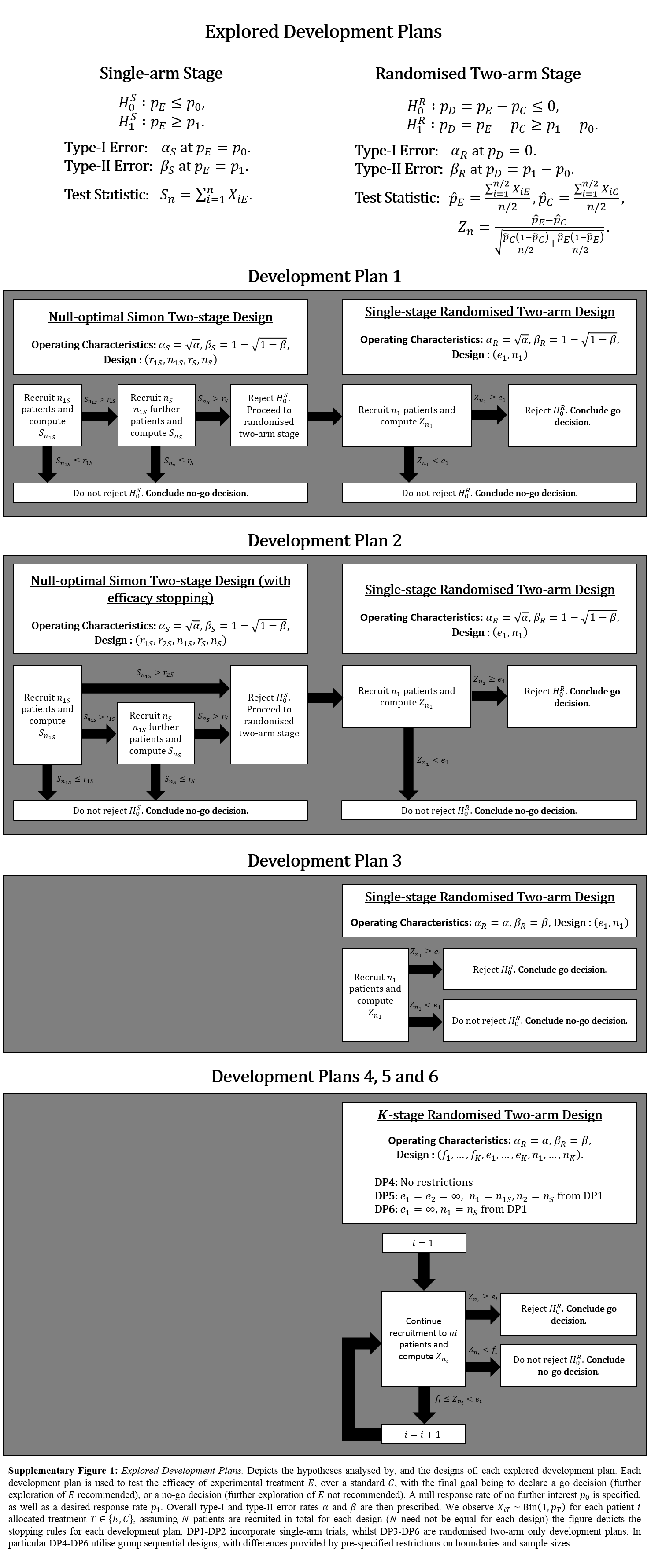

Supplement: Supplementary file 2 — Supporting info item [file PST-15-143-s002.tif]
